# Complete Genome Sequence of a Clinical Isolate of *Enterobacter asburiae*

**DOI:** 10.1128/genomeA.00523-16

**Published:** 2016-06-09

**Authors:** Feng Liu, Jian Yang, Yan Xiao, Li Li, Fan Yang, Qi Jin

**Affiliations:** MOH Key Laboratory of Systems Biology of Pathogens, Institute of Pathogen Biology, CAMS and PUMC, Beijing, P.R. China

## Abstract

We report here the complete genome sequence of *Enterobacter asburiae* strain ENIPBJ-CG1, isolated from a bone marrow transplant patient. The size of the genome sequence is approximately 4.65 Mb, with a G+C content of 55.76%, and it is predicted to contain 4,790 protein-coding genes.

## GENOME ANNOUNCEMENT

*Enterobacter asburiae* belongs to the *Enterobacteriaceae* family. *Enterobacter asburiae* was described and named in 1986 in honor of Mary Alyce Fife-Asbury, an American bacteriologist who made many important contributions to the classification of *Enterobacteriaceae* (1).

*Enterobacter asburiae* are Gram-negative, oxidase-negative, fermentative, and nonpigmented rods with the general characteristics of the family *Enterobacteriaceae* and of the genus *Enterobacter*. It is susceptible to gentamicin and sulfadiazine and resistant to ampicillin, cephalothin, and penicillin. The most closely related species were *Enterobacter cloacae* and *Enterobacter hormaechei* (2).

*Enterobacter* spp. have increasingly been identified as pathogens over the past several decades. Most strains of *Enterobacter asburiae* have been identified and characterized as opportunistic pathogens (3–5), having been isolated from environmental and clinical specimens, such as soil, water, and a variety of human sources, including urine, respiratory tracts, stools, blood, and so on (2, 3, 6).

Whole-genome sequence analysis enables the knowledge of antibiotic resistance, virulence determinants, and the different pathogenic effects of the clinical isolates in different models of infection (7). Therefore, we sequenced the complete genome of *Enterobacter asburiae* strain ENIPBJ-CG1, which was isolated from the bloodstream of a bone marrow transplant patient in a Beijing Hospital.

Genomic DNA was isolated from bacteria using the Qiagen QIAamp DNA minikit (Qiagen, Hilden, Germany). The sequencing reads were sequenced both by IonTorrent PGM using a 314v2 chip and a 400-bp sequencing kit, and by PacBio (Pacific Biosciences, Menlo Park, CA, USA) single-molecule real-time sequencing technology (Duke University, Durham, NC, USA) (8). IonTorrent PGM reads were aligned to the assembled genome of PacBio RS using the Burrows-Wheeler algorithm (9), and sequence discrepancy were then identified using SAMtools (10). The genome was annotated using the NCBI Prokaryotic Genome Annotation Pipeline (http://www.ncbi.nlm.nih.gov/genome/annotation_prok). The *E. asburiae* strain ENIPBJ-CG1 genomer consists of a single chromosome that is 4,648,696 bp in length with a G+C content of 55.76%. The genome contains 4,790 genes assigned with a protein-encoding function, 87 tRNA genes, and 25 rRNA genes organized into 8 rRNA operons.

### Nucleotide sequence accession number.

The complete genome sequence of *E. asburiae* strain ENIPBJ-CG1 has been deposited in GenBank under the accession number CP014993.
